# The Influence of Polymer and Ion Solvation on the Conformational Properties of Flexible Polyelectrolytes

**DOI:** 10.3390/gels4010020

**Published:** 2018-03-02

**Authors:** Alexandros Chremos, Jack F. Douglas

**Affiliations:** Materials Science and Engineering Division, National Institute of Standards and Technology, Gaithersburg, MD 20899, USA

**Keywords:** polyelectrolytes, gels, solvation

## Abstract

The study of the coupling between the conformational properties of a polyelectrolyte chain and the distribution of counter-ions surrounding the chain is important in developing predictive theories for more complex polymer materials, such as polyelectrolyte gels. We investigated the influence of solvent affinity to counter-ions and the polyelectrolyte backbone on the conformational properties of highly charged flexible polymer chains using molecular dynamics simulations that include both ions and an explicit solvent. We find that the solvation of the polyelectrolyte backbone can be achieved by either increasing the solvent affinity for the polyelectrolyte segments or by increasing the solvent affinity for the counter-ions. However, these two mechanisms influence the conformational properties of the polyelectrolyte chain in rather different ways, suggesting the inadequacy of polyelectrolyte solution models that treat the solvent as a continuum medium.

## 1. Introduction

Polyelectrolyte gels consist of charged polymers associated with each other in solution by either physical or chemical bonds, where the polymer–polymer interactions that govern the structure of such material are mediated by counter-ions and solvent [1]. Cross-linked gels have attracted considerable technological and scientific attention due to their capacity to swell in volume by several orders of magnitude compared to their dry volume [2]. Such gels can then be used as superadsorbants in hygiene products [3] and biomedicine [4,5,6], as well as in agriculture to enhance the retention of water in dry soils and as carrier material for releasing agrochemicals with improved efficacy [7,8]. While prior work has mainly focused on polyelectrolyte gels having chemical cross-links, the formation of physical gel through polyelectrolyte association [1,9,10,11] is an equally important class of polyelectrolyte gels that is ubiquitous in biological systems [12,13]. The development of theories and models to predict the swelling behavior, structure, and dynamics of charged polyelectrolyte materials is a challenging problem due to the coupling between the conformational properties of the polyelectrolyte chain and the distribution of counter-ions surrounding the chains. We argue here that a missing “piece of the puzzle” is that theories and simulation models normally treat the solvent as a continuum medium, thus neglecting both the solvation of the ions and the polymeric species. It is our view that continuum treatments are simply not sufficient to capture many of the properties of polyelectrolyte solutions and gels. The present work explores how the solvent interactions with different charged species influence the conformational properties of polyelectrolytes in solution. We restrict our attention in the present work to a single polyelectrolyte chain in solution containing explicit ions and solvent.

Typical polyelectrolyte solution and gel models are described by an extension of the “primitive model” [14,15,16,17] of ionic solutions, where all charged species are treated explicitly as charged hard spheres and the solvent enters the model through its influence on the permittivity of the continuum fluid surrounding the charged species. This is a natural extension of the Debye–Huckel theory [18] of ionic solutions, where the charged species are non-polymeric. In variations of the primitive model (for example, see [19,20,21] for polyelectrolyte solutions and [22,23,24] for polyelectrolyte cross-linking gels), the solvent may also influence the properties of polyelectrolyte systems via effective short-range interactions between the polymer segments, thus mimicking the solvent quality. However, the influence of the solvent interactions for the ionic species in the polyelectrolyte solutions and gels are neglected with this type of continuum solvent models. To illustrate the importance of these interactions, we offer the following example. For typical electrostatic conditions in dilute polyelectrolyte concentrations, a fraction of the counter-ions are condensed along the polyelectrolyte backbone, while the rest of the counter-ions are located at larger distances but remain associated with the polyelectrolyte chain in the form of an ionic cloud surrounding the chains. These two counter-ion “states” are transient, and there is a constant dynamic exchange of counter-ions between them [25,26,27]. This two-state concept for counter-ions is widely accepted in the literature (theoretical [28,29,30,31,32,33,34,35] and experimental studies [25,36,37,38,39,40]), and we have repeatedly observed that solvation influences the structure and dynamics of polyelectrolyte solutions and, by extension, the swelling capacity and response of polyelectrolyte gels. According to the “primitive polyelectrolyte model” and its variations, the solvation of the polyelectrolyte chain is the result of a balance between the tendency of the counter-ions to condense along the polyelectrolyte backbone and residual electrostatic interactions between the counter-ions. However, this balance can be disturbed if the solvent particles have attractive interactions that are strong enough in comparison to the electrostatic interactions between the counter-ions and polyelectrolyte segments; this effect reduces the number of condensed counter-ions along the chain backbone. This simple observation is missing from previous theoretical frameworks of polyelectrolyte solutions and gels, and it is expected to become prominent in conditions where the long-ranged electrostatic interactions between charged species are equivalent to the short-ranged dispersion interactions between the solvent and charged species. In other words, polyelectrolyte and ion solvation results from a *balance* between the competitive associative interactions of three species, namely the counter-ions, the polyelectrolyte segments, *and* the solvent molecules. Competitive binding of molecular species to macromolecules is known to greatly alter the phase behavior of polymer solutions [41,42], leading to often counter-intuitive phase behavior, and an even greater complexity is to be expected when associated species have long-range interactions.

Why does the solvation of ionic species matter so much in polyelectrolyte solutions and gels? We momentarily shift our focus to the relative “simple” case of electrolyte solutions (with no polyelectrolyte chains) to underscore the significance of solvent-ion interactions. It is well known that, in electrolyte solutions with different salts, one can observe a wide spectrum of changes in solution properties, such as density, viscosity, and surface tension; these changes in solution properties are typically classified in terms of the Hofmeister series. Recent observations of Collins [43,44], and theoretical arguments by Ninham et al. [45], suggest the importance of ion size with respect to the extent of ion-solvation and the dispersion interaction between ions and water, respectively, in understanding the trends of polymer solubility, i.e., the Hofmeister series. Indeed, the ion solvation energy effectively reflects a combination of Coulombic and dispersion interaction contributions between the ions and the solvent particles surrounding the ions [46]. Motivated by these observations, we explored an explicit electrolyte solvent model in which the water–ion dispersion interaction parameter was determined by the ion solvation energy through the application of Born theory of ionic solvation [47]. We found that molecular dynamics simulations utilizing this model captured semi-quantitatively observed changes in solution viscosity and water diffusion coefficient on ion type [47], an effect that classical coarse-grained pair-potential models fail to reproduce [48]. Recent calculations of the same model reveal that several other thermodynamic properties, including the density, isothermal compressibility, and surface tension, can be understood via the solvent-ion interactions, suggesting that the Hofmeister series is closely related to ion solvation [49]. Thus, if the solvent interactions with the ionic species plays such a crucial role in modulating the electrolyte solution properties, then it is logical to expect analogous effects in polyelectrolyte solutions and gels.

**Figure 1:**
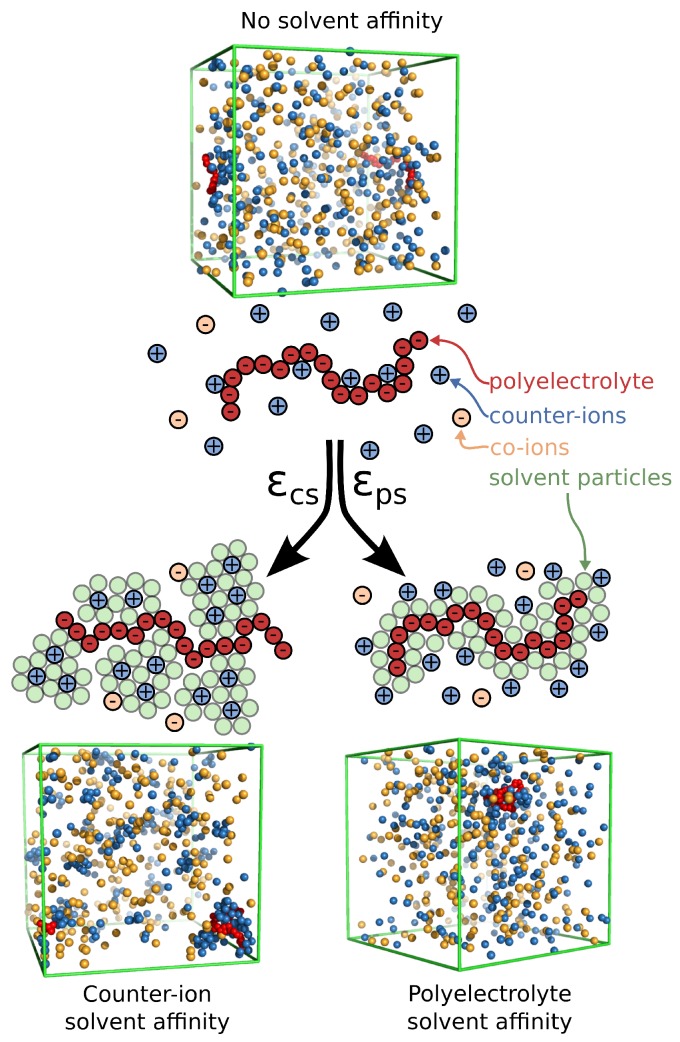
Schematic of a polyelectrolyte chain along with the surrounding ions and the two types of solvent affinity, namely polyelectrolyte solvent affinity originating from strong attractive interactions between the polymer segments and the solvent particles ($\varepsilon_{\mathrm{ps}}$) and the counter-ion solvent affinity originating from the strong attractive interactions of the solvent for the counter-ions ($\varepsilon_{\mathrm{cs}}$). In the schematic figures, the solvent particles that interact strongly with the corresponding ionic species are rendered visible to illustrate how the solvent affinity influences the solution structure in close proximity to the polyelectrolyte chain. Typical screenshots of the system for each solvent affinity type is also presented; the solvent particles at the screenshots are rendered invisible for clarity.

To probe and quantify the influence of the solvent interactions with the ionic species, we focus on the simplest case of polylectrolyte solution systems, an isolated polyelectrolyte chain in solution corresponding to a highly dilute polymer concentration. Our motivation is also drawn from our previous work [11], where we show that counter-ion solvation can lead to effective long-range attractive interchain interactions in salt-free polyelectrolyte solutions that can also greatly influence thermodynamic and dynamic properties of these polymer solutions. The choice of this type of system allows us to examine the solvent effects independently of the polymer network architecture and/or polymer concentration effects. We utilize a computational model that incorporates minimal aspects of a real polyelectrolyte solution and, at the same time, allows for equilibrated molecular dynamics with an explicit solvent, see Figure 1. The added cost in computational time required for the inclusion of an explicit solvent is necessary to fully capture the polyelectrolyte structural and dynamical behavior, as indicated by a series of studies [11,50,51,52,53]. In particular, an explicit solvent is necessary in order to study the variation of the solvent interactions with different ionic species. In particular, we focus on the changes in the conformational properties of the polyelectrolyte chain and the distribution of the counter-ions surrounding the polymer for two types of solvent affinity, namely the solvent affinity for the counter-ions and the solvent affinity for the polyelectrolyte chain.

Our paper is organized as follows. Section 3 contains details of the model and simulation methods. Results of the conformational properties of the polyelectrolyte chain and the characterization of the spatial distribution of the counter-ions surrounding the polyelectrolyte chain are presented in Section 2. Section 4 concludes the paper.

## 2. Results and Discussion

**Figure 2:**
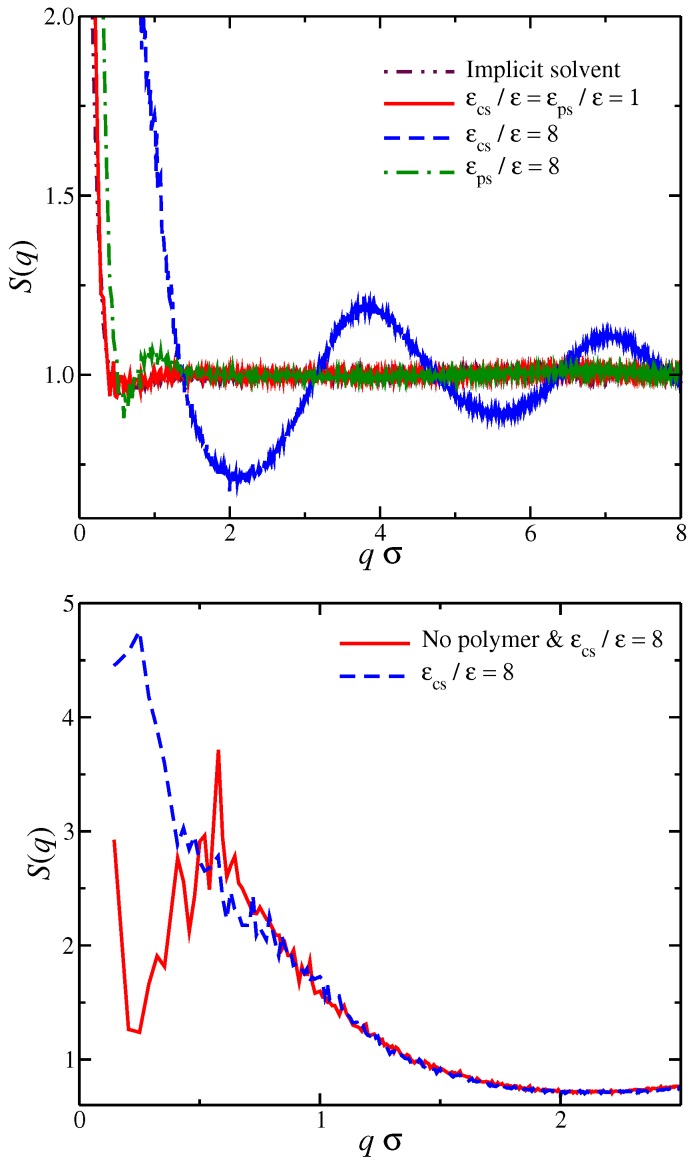
(**Top**) Structure factor S(q) of all counter-ions in the solution. Results for different types of solvent affinity are also presented. Specifically, polyelectrolyte solvent affinity originating from strong attractive interactions between the polymer segments and the solvent particles ($\varepsilon_{\mathrm{ps}}$) and the counter-ion solvent affinity originating from the strong attractive interactions of the solvent for the counter-ions ($\varepsilon_{\mathrm{cs}}$). For comparison, we also include the results of the solvent with no affinity ($\varepsilon_{\mathrm{cs}}/\varepsilon =\varepsilon_{\mathrm{cs}}/\varepsilon =1$) and the results for the implicit solvent model. (**Bottom**) S(q) of all counter-ions in a solvent having strong solvent affinity for the counter-ions $\varepsilon_{\mathrm{cs}}/\varepsilon =8$ with and without the polyelectrolyte chain.

Before we begin discussing the conformational properties of the polyelectrolyte chain, we briefly examine the spatial correlations between the counter-ions at different types of affinity solvent. The static structure factor, S(q), is a suitable property for this purpose and describes the mean correlations in the positions for a collection of point particles (in our case, the positions of all the counter-ions), S(q):

(1) $$S\left(q\right)=\frac{1}{N_{+}}\langle \sum_{j=1}^{N_{+}}\sum_{k=1}^{N_{+}}\exp\left[-iq\cdot (r_{j}-r_{k})\right]\rangle ,$$

where $i=\sqrt{-1}$, q=|q| is the wave number, $r_{j}$ is the position of particle *j*, and 〈〉 denotes the time average. For a solvent having no preferential affinity ($\varepsilon_{\mathrm{cs}}/\varepsilon =\varepsilon_{\mathrm{ps}}/\varepsilon =1$), we find that S(q)≈1 for qσ>1, which means that, for these length scales, there is no spatial correlation between the counter-ions, see Figure 2. For qσ<0.8, there is an upturn in S(q), which means that there are significant density fluctuations in these length scales. Typically, a significant excess scattering in low *q*-regime means one of two things: Either a macrophase separation or the formation of clusters in the system. A visual inspection of the system (Figure 1) clearly indicates that the latter is taking place rather than the former. The S(q) behavior between solvents having no preferential affinity and the case of implicit solvent is very similar, indicating that the spatial correlations of the ions in these two cases are very similar. For solvents having strong affinity for the polyelectrolyte segments, we find that there is a small oscillatory behavior at length scales on the order of the chain size and no correlations at qσ>1, which is similar to solvents with no affinity. We interpret this behavior as follows. An increase in $\varepsilon_{\mathrm{ps}}$ leads to the formation of a more compact solvation layer surrounding the polyelectrolyte chain, thus inhibiting its mobility. This localization of polyelectrolyte segments influences the localization of nearby counter-ions as observed in S(q). For solvents having a strong affinity for the counter-ions, we find that there are fluctuations in all length scales. In particular, we find that there is significant excess scattering compared to the other two types of solvent affinities in the low *q*-regime. Moreover, for qσ>1, there are sinusoidal-like oscillations in S(q) behavior, suggesting the existence of charge density waves that become damped at large distances similar to the local ordering in dense fluids. We interpret all these observations as follows: the strong counter-ion solvation leads to the formation of counter-ion clusters having a liquid-like structure, while there are large ion density fluctuations at length scales larger than the cluster size. We note that these features in counter-ion affinity solvents exist even in the absence of polyelectrolyte chains, and the presence of the polyelectrolyte chains influences the structure of the counter-ions at a low *q*-region, as seen in Figure 2. We thus see a significant deviation from the physical picture indicated by the polyelectrolyte primitive model once we explicitly consider the solvent and its differential interaction with different ionic species.

Now that we have a basic understanding on how the different types of solvent affinities influence the structure of the ions in an electrolyte solution, we focus on the characterization of the interfacial layer around the polyelectrolyte backbone. Theoretically, correlations between the counter-ions and the polyelectrolyte chains are usually described based on the classical counter-ion condensation theory of Manning and subsequent revisions of this classic model of polyelectrolytes [54,55,56]. According to this theory, the counter-ions from their uniform distribution in the solution start to “condense” on the chain backbone, when the electrostatic interactions become comparable to thermal energy. This instability takes place when $\xi =\lambda l_{B}>1$ (λ is the polyelectrolyte charge per length). However, the Manning theory models polyelectrolytes as infinitely long charged straight threads, while real polyelectrolytes have a finite chain length and can be relatively flexible. The existence of a flexible backbone raises basic and theoretically unresolved questions about how the polyelectrolyte conformation influence the distribution of counter-ions around the polyelectrolyte chain and how these counter-ions, in turn, influence polymer conformation.

**Figure 3:**
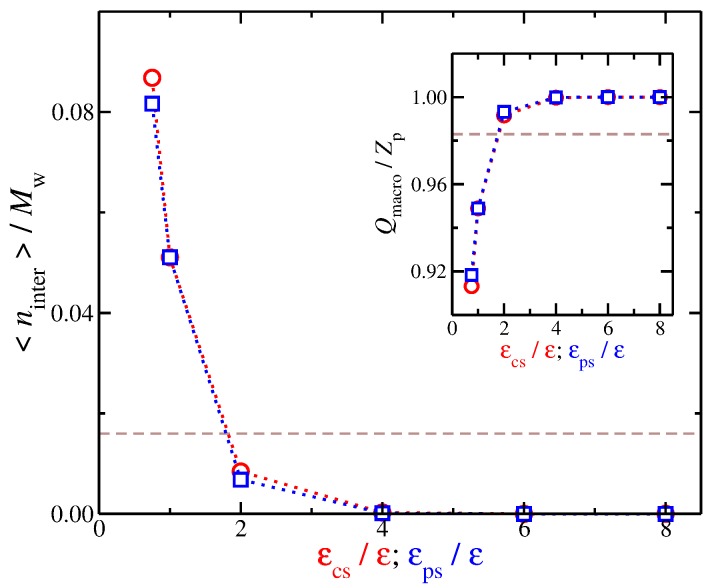
Time average number of interfacial counter-ions $\langle n_{\mathrm{inter}}\rangle$ normalized by the molecular mass as a function of the strength of solvent affinity. Specifically, the solvent affinity for the counter-ions $\varepsilon_{\mathrm{cs}}$ (cicles), and the solvent affinity of the polyelectrolyte segments $\varepsilon_{\mathrm{cs}}$ (squares). The dashed line corresponds to the implicit solvent model. Inset: Effective polyelectrolyte charge, $Q_{\mathrm{macro}}=Z_{p}-\langle n_{\mathrm{inter}}\rangle$, as a function of the strength of the cross-energy interaction parameters. The uncertainty estimates are smaller than the symbol size.

We set the interfacial layer based on an arbitrary distance criterion in which any counter-ion that is located at shorter distances than 1.1σ are taken to be part of the interfacial region. This particular value, i.e., 1.1σ, is chosen in order to discriminate between the counter-ions that are in contact with the polyelectrolyte from the remaining counter-ions. Based on our model, we find that the interfacial counter-ions exhibit a rich spectrum of behaviors for the different molecular topologies [27] and counter-ion valance [52]. Now that we have defined the interfacial region for our model, we calculate the time average number of interfacial counter-ions, $\langle n_{\mathrm{inter}}\rangle$, for different values of $\varepsilon_{\mathrm{pc}}$. As seen in Figure 3, $\langle n_{\mathrm{inter}}\rangle$ decreases as the strength of the solvent affinity for counter-ions and for the polyelectrolyte segments, e.g., for solvents having either $\varepsilon_{\mathrm{cs}}/\varepsilon >4$ or $\varepsilon_{\mathrm{ps}}/\varepsilon >4$ increases, and we find $\langle n_{\mathrm{inter}}\rangle \approx 0$. In other words, both types of solvent affinity for the charged species result in this effect; the solvent particles are effectively “kicking out” the counter-ions from the polyelectrolyte backbone. We also note that these interfacial counter-ions screen a significant portion of the bare charge of the polyelectrolyte due to their close proximity to polyelectrolyte. We calculate the effective polyelectrolyte charge as $Q_{\mathrm{macro}}=Z_{p}-\langle n_{\mathrm{inter}}\rangle$ and, as expected, the decrease of the number of interfacial counter-ions leads to the ionization of the chain backbone, as can be seen in the inset of Figure 3. These two types of solvent affinities exhibit approximately the same trends from the standpoint of interfacial counter-ions, so it leads us to to consider the question of whether there are any qualitative differences between these two solvent affinities.

**Figure 4:**
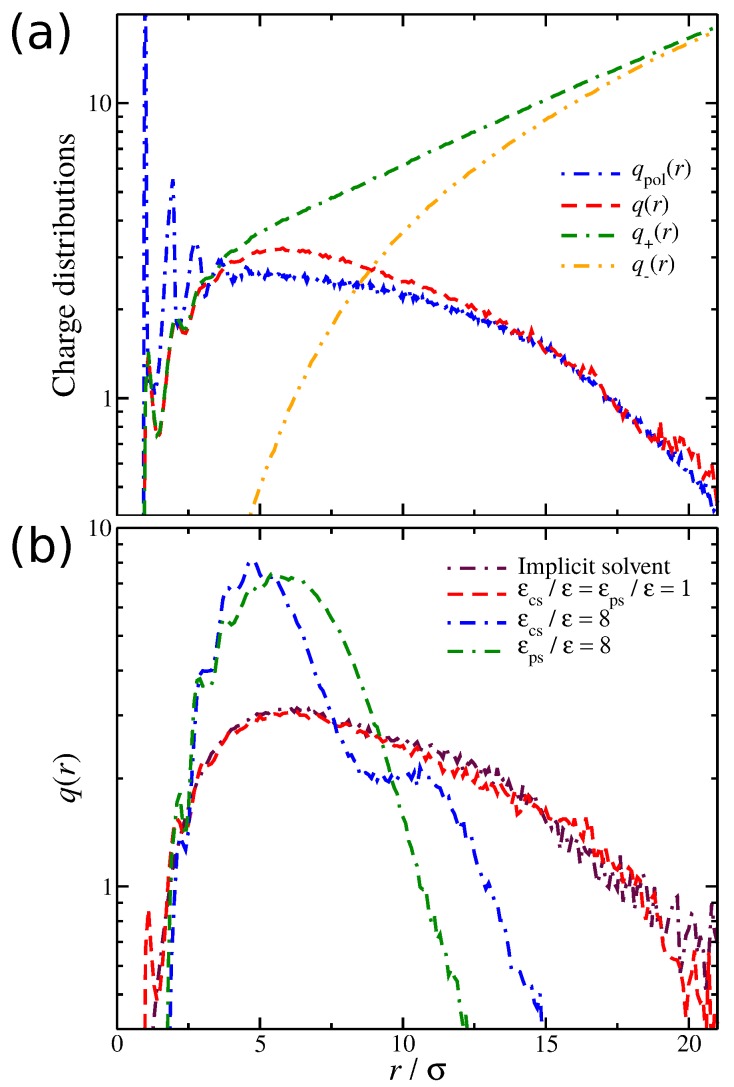
(**a**) Distribution of the ionic net charge, q(r), as well as the relevant distributions of the counter-ions $q_{+}\left(r\right)$ and co-ions $q_{-}\left(r\right)$, and the distribution of the polymeric segments, $q_{\mathrm{pol}}\left(r\right)$, as a function of the distance from the polyelectrolyte segments. The results are obtained for the case of the solvent with no affinity, i.e., $\varepsilon_{\mathrm{cs}}/\varepsilon =\varepsilon_{\mathrm{ps}}/\varepsilon =1$. (**b**) Distribution of the net charge q(r) for three different types of solvent affinity, namely, no solvent affinity ($\varepsilon_{\mathrm{cs}}/\varepsilon =\varepsilon_{\mathrm{ps}}/\varepsilon =1$), strong counter-ion solvent affinity ($\varepsilon_{\mathrm{cs}}/\varepsilon =8$), and strong polyelectrolyte solvent affinity ($\varepsilon_{\mathrm{ps}}/\varepsilon =8$). Results for different solvent affinities, namely (top) between the solvent and positive ions $\varepsilon_{\mathrm{cs}}$ and (bottom) between the solvent and polyelectrolyte segments.

We now focus on the spatial distribution of counter-ions in relation to the position of the polyelectrolyte segments. Previously, we developed an approach for quantifying the spatial distribution of the counter-ions surrounding a polyelectrolyte chains, and we briefly outline this approach [27,52]. In particular, we calculate the average net charge q(r) as a function ofthe distance from the polyelectrolyte segments. This calculation is based on the construction of a histogram that calculates the time average ion charge (counter-ions and co-ions) as a function of the distance from the polyelectrolyte segments; the histogram includes information from all polyelectrolyte segments. As shown in Figure 4a, q(r) is simply the difference of the counter-ion distribution $q_{+}\left(r\right)$ and the co-ion distribution $q_{-}\left(r\right)$, meaning that q(r) contains information for the counter-ions that are located both in the interfacial layer (defined as any particle being at a distance r/σ≤1.1 from any polyelectrolyte segment) and in the diffuse counter-ion cloud. This approach [27,52] allows us to determine the size of the cloud of the diffuse counter-ions ($R_{\mathrm{cloud}}$) associated with the polyelectrolyte chain, since the boundary between this cloud and the bulk is at $q\left(r=R_{\mathrm{cloud}}\right)=q_{+}\left(r\right)-q_{-}\left(r\right)=0$. An example illustrating these charge distributions is presented in Figure 4a. For a weak dispersion interaction strength, $\varepsilon_{\mathrm{cs}}/\varepsilon =1$, a fraction of counter-ions has a slight tendency to “condense” along the polyelectrolyte backbone. However, as we increase the solvent affinity for either the counter-ions or the polyelectrolyte segments, we decrease the average number of condensed counter-ions along the polyelectrolyte backbone, thus altering the q(r) distribution, as illustrated in Figure 4b. The newly dissolved counter-ions continue to interact with the polyelectrolyte chain at relative large distances 2<r/σ<6, leading to an enrichment of the diffuse counter-ion cloud surrounding the polyelectrolyte chain. While the $\langle n_{\mathrm{inter}}\rangle$ trends between the two different types of solvent affinities are approximately the same, the q(r) curves exhibit qualitative deviations from each other. These differences are related to the different molecular conformation that the polyelectrolyte chain is adopting, which we discuss in more detail below.

**Figure 5:**
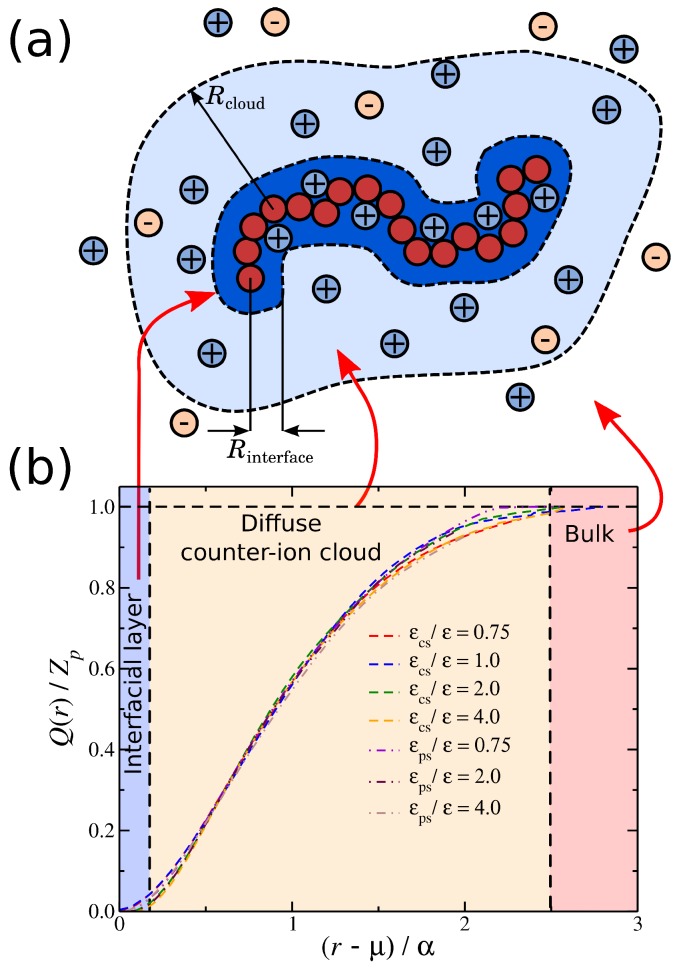
(**a**) A schematic of a polyelectrolyte chain surrounded by ions. The shaded areas highlight the interfacial ions (dark blue) and the domain ions (light blue). Any ions outside these areas correspond to undisturbed ionic solvent. (**b**) Cumulative distribution of the net ionic charge Q(r) around a polyelectrolyte chain normalized by the total polyelectrolyte bare charge $Z_{p}$ for different solvent affinities. Specifically, the solvent affinity for the counter-ions $\varepsilon_{\mathrm{cs}}$ (dashed lines) and the solvent affinity of the polyelectrolyte segments $\varepsilon_{\mathrm{cs}}$ (dot-dashed lines). The shaded areas correspond to different types of ions depending on their distance from the polyelectrolyte segments.

To better characterize these charge distributions, we consider the *cumulative net charge*, $Q\left(r\right)=\int_{0}^{r}q\left(x\right)dx$ at a distance *r* from macro-ion segments. As we have discussed in previous work [27,52], Q(r) quantifies the net ionic distribution around a polyelectrolyte chain. A basic feature of Q(r) is that it starts from 0 at short distances r/σ<1 and progressively increases at long distances until it saturates, i.e., $Q\left(r\right)/Z_{p}\approx 1$, see Figure 5. The rate at which $Q\left(r\right)/Z_{p}$ reaches unity seems to follow the approximately universal functional form:

(2) $$Q\left(r\right)=Z_{p}\tanh^{2}\left[(r-\mu )/\alpha \right]$$

where α and μ are fitting parameters. The values of the μ parameter are of the order unity, where μ indicates the location at which Q(r) starts to progressive increase.

**Figure 6:**
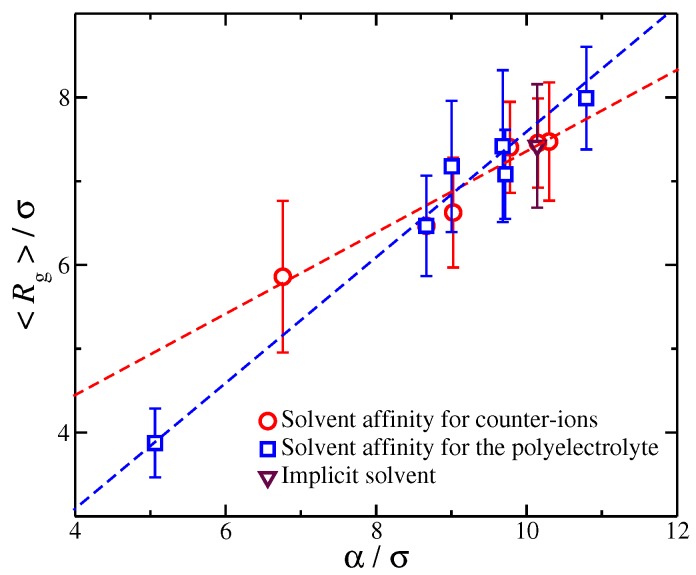
The relationship between α parameter characterizing the size of the ionic cloud around the polyelectrolyte chains and the average radius of gyration $R_{g}$ of the polyelectrolyte chain. Each point corresponds to a different strength solvation affinity for the charged species. The dashed line are fits for the different solvent affinities for the ionic species and the error bars correspond to two standard deviations.

This suggests that μ is associated with the amount of charge in close proximity to the polyelectrolyte backbone. For the purpose of our study, we focus on the size α, since α determines the overall size of the diffuse counter-ion cloud. We note that we previously found that the functional form of Equation (2) held for polyelectrolytes having different molecular architectures [27], and this relation held even for for different counter-ion valence [52], suggesting that the rate of charge saturation is generally *coupled* with the structure of the polyelectrolyte chains and the charge carried by the counter-ions. Moreover, for monovalent counter-ions, the size of ionic cloud is directly coupled with the size of the polyelectrolyte chain, as quantified by the radius of gyration, $R_{g}$ [27]. Here, we extend this type of calculation to polyelectrolyte chains having different degrees of solvent affinity. For each value of the strength of the solvent affinity parameter $\varepsilon_{\mathrm{ps}}$ or $\varepsilon_{\mathrm{cs}}$, we expect to influence the value of the time average $R_{g}$ of the polyelectrolyte chain. Thus, the following question arises: Is the size of the ionic cloud, coupled with the changes in the size of the polyelectrolyte chains, induced by the changes in the strength of the solvent affinity? By plotting the time average $\langle R_{g}\rangle$ as a function of α, we find that the average size of the polyelectrolyte chain with is again found to scale with the α-parameter as we vary with the solvent affinity for the ionic species, see Figure 6. This finding agrees with our observations from our previous study where we examined the impact of molecular architecture on the size of the counter-ion cloud [27]. It is worth pointing out that, while we find $\langle R_{g}\rangle \propto \alpha$ for both types of solvent affinities, the prefactor of this linear relation are different between the two solvent affinities. Similar deviations from the monovalent counter-ions were also found for divalent and trivalent counter-ions, where the trend was amplified due to the stronger coupling between counter-ions with the conformational properties of the polyelectrolyte chain, leading to a non-trivial dependence between the size of the ionic cloud and $R_{g}$ [52]. Thus, the solvation layer around different charged species influences this coupling in a non-trivial way.

**Figure 7:**
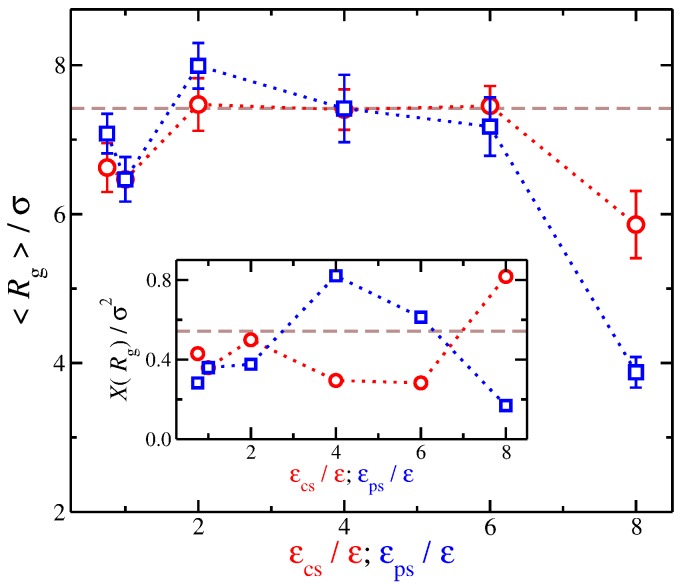
Average radius of gyration $\langle R_{g}\rangle$ of the polyelectrolyte chain as a function of the strength of solvent affinity for the counter-ions ($\varepsilon_{\mathrm{cs}}$) and the polyelectrolyte segments ($\varepsilon_{\mathrm{ps}}$). The symbols represent solvents with affinity for the counter-ions (circles) and polyelectrolyte segments (squares). The error bars correspond to one standard deviation. Inset: Variance of $R_{g}$, $X\left(R_{g}\right)$, as a function of $\varepsilon_{\mathrm{cs}}$ and $\varepsilon_{\mathrm{ps}}$. The dashed lines correspond to the implicit solvent case.

Our findings regarding the relation between $R_{g}$ and α suggest that we examine the dependence of $R_{g}$ on solvent affinity. We find qualitatively similar trends from a quick look at the resulting average values of $R_{g}$ with variation in the strength of the different types of solvent affinities. Nevertheless, a quantitative comparison reveals additional differences between the different types of solvent affinity. In particular, we found that, while an increase from $\varepsilon_{\mathrm{cs}}/\varepsilon =1$ to 2 leads to an increase in $R_{g}$, a stronger degree of swelling occurs if $\varepsilon_{\mathrm{ps}}$ increases by the same amount. While the time average $R_{g}$ remains approximately constant for solvents having a counter-ion affinity ($2<\varepsilon_{\mathrm{cs}}/\varepsilon <6$), for solvents having polyelectrolyte affinity, there is a monotonic decease of $\langle R_{g}\rangle$ with $\varepsilon_{\mathrm{ps}}$. Even while the time average value of $R_{g}$ ($\langle R_{g}\rangle$) is approximately the same for both solvent affinities, i.e., solvents having $\varepsilon_{\mathrm{cs}}/\varepsilon =4$ and solvents having $\varepsilon_{\mathrm{ps}}/\varepsilon =4$, the variance of $R_{g}$, labeled as $X(R_{g})$, between these solvents is significantly different, see the inset of Figure 7. Specifically, $X(R_{g})$ for polyelectrolyte affinity solvents having $\varepsilon_{\mathrm{ps}}/\varepsilon =4$ is larger than that for counter-ion affinity solvents having $\varepsilon_{\mathrm{cs}}/\varepsilon =4$ by a factor of two. In other words, while the time average $R_{g}$ is approximately the same, the fluctuations can be significantly different reflecting the influence of the solvent between the charged species.

**Figure 8:**
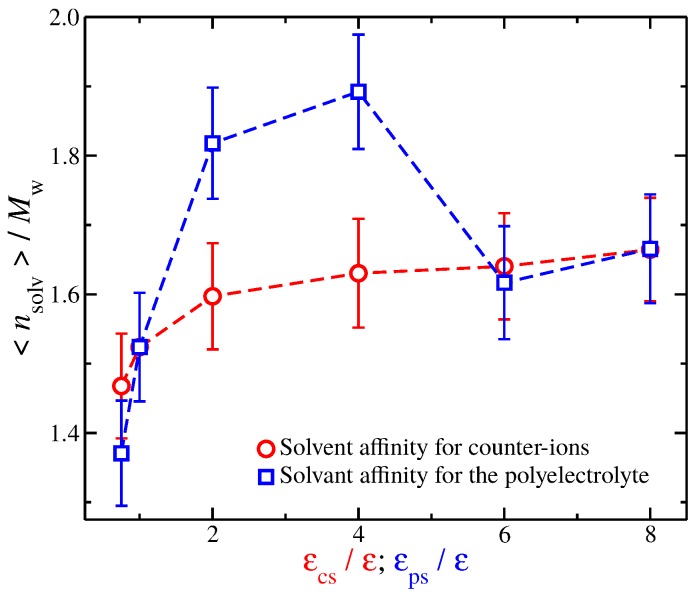
Average number of interfacial solvent particles $\langle n_{\mathrm{solv}}\rangle$ along the polyelectrolyte backbone normalized by the molecular mass $M_{w}$ as a function of the strength of solvent affinity for the counter-ions ($\varepsilon_{\mathrm{cs}}$) and the polyelectrolyte segments ($\varepsilon_{\mathrm{ps}}$). The symbols represent solvents with affinity for the counter-ions (circles) and polyelectrolyte segments (squares). The error bars correspond to two standard deviations.

A closer examination at the solvation layer is required to better understand the similarities and differences in $R_{g}$ in Figure 7. For this reason, we calculate the time average number of interfacial solvent particles $\langle n_{\mathrm{solv}}\rangle$ that are in contact with the polyelectrolyte backbone. A solvent particle is in contact with a polyelectrolyte segment if it is located at a distance r≤1.1σ. The results are presented in Figure 8. In particular, we find that, for solvents with weak affinities for the charged species (solvents having $\varepsilon_{\mathrm{cs}}/\varepsilon ≲1$ and for solvents having $\varepsilon_{\mathrm{ps}}/\varepsilon ≲1$), the $\langle n_{\mathrm{solv}}\rangle$ values are similar. The same trend is found for strong solvent affinity for the charged species (solvents having $\varepsilon_{\mathrm{cs}}/\varepsilon ≳6$ and for solvents having $\varepsilon_{\mathrm{ps}}/\varepsilon ≳6$). However, for solvents with intermediate affinities, we find considerable differences between the two types of solvents. Specifically, solvents having $1<\varepsilon_{\mathrm{ps}}/\varepsilon <6$ have significantly more solvent particles than solvents having $1<\varepsilon_{\mathrm{cs}}/\varepsilon <6$. This is not surprising since enhancing the cross energy interaction parameter between solvent particles and polyelectrolyte segments results in a “tight and sticky” packing of the solvent particles around the polyelectrolyte segments. This effect is more noticeable in the comparison between a solvent having $\varepsilon_{\mathrm{cs}}/\varepsilon )=4$ and a solvent having $\varepsilon_{\mathrm{ps}}/\varepsilon )=4$ because the $R_{g}$ is approximately the same, see Figure 7. Nevertheless, we anticipate that the differences in the solvent packing at and near the interfacial layer between the different solvent affinities for the charged species will be found to be responsible for the differences observed in the fluctuations in $R_{g}$ and the shrinkage of polymer size for strong solvent affinity for the charged species.

**Figure 9:**
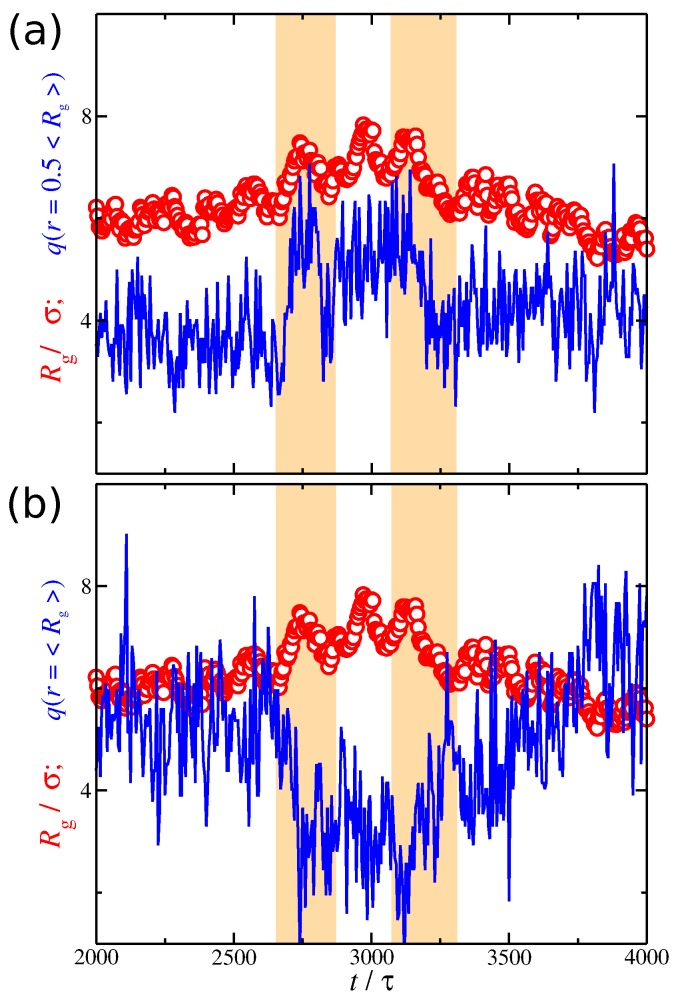
Radius of gyration $R_{g}$ of the polyelectrolyte chain (circles) and the average ionic charge (line) at a given distance from polyelectrolyte segments (**a**) $q(r=0.5\;R_{g})$ and (**b**) $q(r=R_{g})$ as a function of time *t*. The results correspond to a solvent having strong attractive interactions with the counter-ions, i.e., $\varepsilon_{\mathrm{cs}}/\varepsilon =8$. The scale in the *y*-axis is the same for both $R_{g}$ and q(r). The shaded regions are guides for the eye.

So far, we have investigated the conformational properties of the polyelectrolyte chain and the spatial distribution of the counter-ions separately from each other. While we have demonstrated that the conformational properties of the polyelectrolyte chains are correlated with the distribution of the counter-ions, we have not yet directly examined the nature of this correlation. The simplest way to probe their correlation is to plot the time series of $R_{g}$ and q(r) at different distances from the polyelectrolyte segments. Based on Figure 9, it is clear that $R_{g}$ fluctuates at a lower rate than q(r). We anticipate this behavior since the polyelectrolyte chain is a larger and more slowly relaxing molecular species than the smaller and more mobile ions. In the example presented in Figure 9, it is evident that the net charge at $r=\langle R_{g}\rangle /2$ follows closely the changes observed in $R_{g}$, which means that they are positively correlated. However, at larger distances $r=\langle R_{g}\rangle$, $R_{g}$ exhibits the opposite trends of the net charge, meaning that they are *anti-correlated*. However, these correlation effects are not always obvious, as in the example shown in Figure 9. We thus need a better metrology for this phenomenon.

Now that we have determined the average size of the diffuse ionic cloud associated with the polyelectrolyte chain in solution, we can probe the following phenomenon. Past literature studies have generally assumed that salt is evenly distributed between polyelectrolyte gel or a polyelectrolyte complex coacervate in contact with an aqueous phase [57], even though the asymmetry in salt concentration between the polymer rich and the solvent rich phases has been known since the original work of Voorn-Overbeek [58]. This asymmetry can be understood at least conceptually from a rough calculation based on our model. We calculate the density of the ions that are within the diffuse counter-ion cloud as we defined it above (see also Figure 5), assuming that the overall domain is spherical with a radius, $R_{\mathrm{cloud}}=5\alpha /2$, and compare it with the corresponding ion density of an electrolyte solution with the same $l_{B}$ and $\lambda_{D}$ parameters. While a more precise characterization of the volume of the ionic cloud is required for a precise comparison, we find that the ion density in the ionic cloud associated with the polyelectrolyte chain is an order of magnitude larger than that in the corresponding electrolyte solution, as expected.

**Figure 10:**
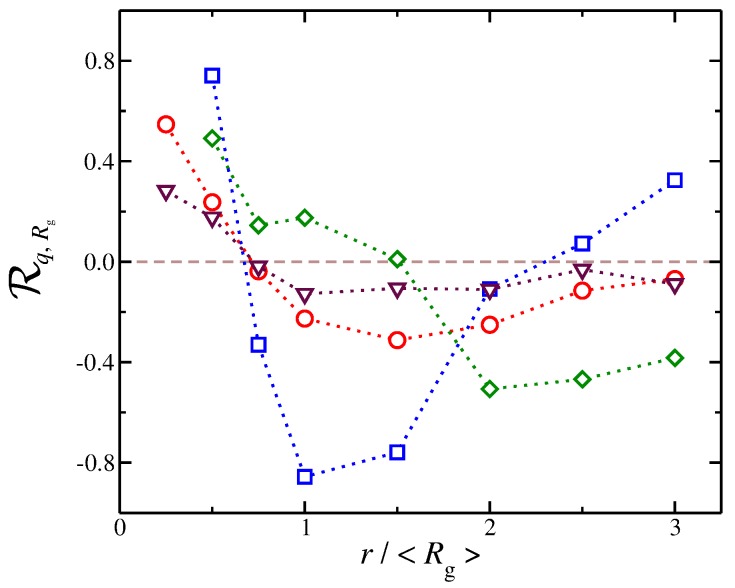
Pearson correlation coefficient $R_{q,R_{g}}$ for the radius of gyration $R_{g}$ and the net ionic charge q(r) as a function of the distance, *r*, from polyelectrolyte segments. Symbols represent systems where there is no solvent affinity $\varepsilon_{\mathrm{cs}}/\varepsilon =\varepsilon_{\mathrm{ps}}/\varepsilon =1$ (red circles) and where there is solvent affinity for counter-ions $\varepsilon_{\mathrm{cs}}/\varepsilon =8$ (blue squares), solvent affinity for polyelectrolyte segments $\varepsilon_{\mathrm{ps}}/\varepsilon =8$ (green diamonds), and implicit solvent (maroon triangles).

We quantify these correlations using the Pearson correlation coefficient [59,60,61]. In particular, we quantify the correlation between the values of $R_{g}$ and the net ionic charge q(r) at different distances from the polyelectrolyte segments. The Pearson correlation coefficient is a measure of the strength of the association between two discrete variables $x_{i}$ and $y_{i}$ and it is defined as,

(3) $$R_{x,y}=\frac{\sum_{i=1}^{n}\left(x_{i}-\langle x\rangle \right)\left(y_{i}-\langle y\rangle \right)}{{\left[\sum_{i=1}^{n}{\left(x_{i}-\langle x\rangle \right)}^{2}\right]}^{1/2}{\left[\sum_{i=1}^{n}{\left(y_{i}-\langle y\rangle \right)}^{2}\right]}^{1/2}}$$

where 〈.〉 denotes the mean value. It has a value between +1 and −1, where 1 is the highest positive correlation, 0 is no linear correlation, and −1 is the highest negative correlation, i.e., an anti-correlation. The counter-ions exhibit different correlations with $R_{g}$ at different distances, see Figure 10. At short distances, we find that $R_{q,R_{g}}$ is positive, meaning that, as $R_{g}$ increases, there is then an increase in q(r), a trend that is observed for all types of solvents that we have explored here. This trend can be understood as follows: When the polyelectrolyte chain is in the process of swelling, i.e., there is an increase in $R_{g}$, it makes “space” for counter-ions to approach the polyelectrolyte backbone, leading to an increase in q(r). At larger distances, we find that $R_{q,R_{g}}$ becomes negative and reaches a minimum. In other words, as the polyelectrolyte chains swells, the amount of net charge at larger distances decreases significantly. This is understandable because the process of swelling includes two competing effects that occur simultaneously and give rise to this anti-correlation effect. First, a fraction of counter-ions from the diffuse counter-ion cloud approach the chain at shorter distances as we described above. Second, as the chain swells, the charge density of the chain becomes reduced, resulting in a smaller repulsion for co-ions in the bulk, which can approach the polyelectrolyte chain at shorter distances. This effect reduces q(r) at distances on the order of the chain size. In this second regime, where the q(r) is anti-correlated with $R_{g}$, we find that different types of solvent result in different trends. For example, a polyelectrolyte implicit solvent model tends to have $R_{q,R_{g}}$ values close to 0, meaning that there is little or no correlation between these two quantities. However, for solvents having a strong affinity for the counter-ions, we observe the sharpest variations in $R_{q,R_{g}}$. Specifically, we find that, at short distances, there is a strong positive correlation $R_{q,R_{g}}\left(r=\frac{1}{2}\langle R_{g}\rangle \right)\approx 0.8$; however, at the largest distances, strong anti-correlations with $R_{q,R_{g}}\left(r=\langle R_{g}\rangle \right)\approx -0.8$ are exhibited.

We observe that the counter-ion distribution for solvent having no affinity and the case of implicit solvent have approximately the same distribution, but $R_{q,R_{g}}$ in the implicit solvent case are smaller than solvents with no affinity. The results presented in Figure 10 highlight that the dispersion of interaction of the solvent with the charged species influence the coupling between the distribution of counter-ions and the conformational properties in subtle ways. These results, as well as the results presented in our study, further reinforce the viewpoint that an explicit solvent in the modeling of polyelectrolyte solutions and gels is necessary to capture the solution properties of polyelectrolyte solutions.

For the purposes of our study, the results in Figure 10 provide a qualitative picture of how solvation can influence the dynamical coupling between the ionic cloud surrounding the polyelectrolyte chains and its conformation. While additional work is required to fully understand the nature of the relation between the solvation and this coupling, our findings point to one conclusion. One can view the changes in the polyelectrolyte chain conformation as the cause of the changes in the distribution of the ions surrounding the polyelectrolyte chain, but at the same time the reverse is also valid, i.e., changes in the distribution of the ions influence the polyelectrolyte conformations. Based on our model, we believe that both sides, i.e., polyelectrolyte conformation and the ionic cloud surrounding the polymer, are continuously interacting each other in a dynamic fashion. Thus, the coupling becomes similar to the causality dilemma, “which came first: the chicken or the egg,” where each side can be considered the cause *and* the effect. Our simulation observations reveal that the solvation of the charged species is an important factor in probing basic and theoretically unresolved questions about how the polyelectrolyte conformation affects the distribution of counter-ions distributed around these polymers and about how the counter-ions, in turn, influence polymer conformation.

## 3. Materials and Methods

We employ a bead-spring model of Lennard–Jones (LJ) segments bound by stiff harmonic bonds suspended in explicit LJ solvent particles, some of which are charged to represent counter-ions [26,50,51]. The system is composed of a total of N=64,000 particles in a periodic cube of side *L* and volume *V*. The system includes a single polyelectrolyte chain having a molecular mass of $M_{w}=41$, and a total charge $-Z_{p}e$ is distributed uniformly along the molecular structure, where *e* is the elementary charge. For the purposes of our investigation, we focus on systems having $Z_{p}/M_{w}=1$. The bonds between polymer segments are connected via a stiff harmonic spring, $V_{H}\left(r\right)=k{\left(r-l_{0}\right)}^{2}$, where $l_{0}=\sigma$ is the equilibrium length of the spring, and $k=1000\varepsilon /\sigma^{2}$ is the spring constant. The system also includes $N_{-}$ co-ions of charge −e and $N_{+}=N_{-}+Z_{p}$ counter-ions of charge +e so that the system of interest has a neutral total charge.

All macro-ion segments, dissolved ions, and solvent particles are assigned the same mass *m*, size σ, and strength of interaction ε. We set ε and σ as the units of energy and length; the cutoff distance for LJ interaction potential is $r_{c}=2.5\sigma$. The size and energy parameters between *i* and *j* particles are set as $\sigma_{ii}=\sigma_{jj}=\sigma_{ij}=\sigma$ and $\varepsilon_{ii}=\varepsilon_{jj}=\varepsilon_{ij}=\varepsilon$), except for two energy interaction parameters: the first interaction parameter is between the solvent particles and the polyelectrolyte segments $\varepsilon_{\mathrm{ps}}$ and the second one is between the solvent particles and the positive ions $\varepsilon_{\mathrm{cs}}$. Variation in the interaction energy parameters between different types of particles reflect the degree of chemical incompatibility between the polymer repeating units [62]. For the purpose of our study, we focus on characterizing the effects of the variation of one interaction parameter separately. For example, when we state that $\varepsilon_{\mathrm{cs}}/\varepsilon =4$, then $\varepsilon_{\mathrm{ps}}/\varepsilon =1$. All charged particles interact via Coulomb potential (with a cut-off distance of 10σ) and a relatively short-range Lennard–Jones potential of strength ε, and the particle–particle particle–mesh method is used [63].

The systems were equilibrated at constant pressure and constant temperature conditions, i.e., reduced temperature $k_{B}T/\varepsilon =0.75$ (where $k_{B}$ is Boltzmann’s constant) and reduced pressure 〈P〉≈0.02, and the production run was performed at a constant temperature and a constant volume, maintained by a Nosé–Hoover thermostat. The Bjerrum length was set equal to $l_{B}=e^{2}/\left(\epsilon_{s}k_{B}T\right)=1.85\sigma$, where $\epsilon_{s}$ is the dielectric constant of the solvent. We note that, while we have introduced an LJ fluid as our explicit solvent into our model, it does not influence the nature of electrostatic interactions, so we tune them via the dielectric constant. The Debye screening length is expressed as follows: $\lambda_{D}={\left[4\pi l_{B}\left(\rho_{+}+\rho_{-}\right)\right]}^{-1/2}\approx 2.4$, where $\rho_{\pm }=N_{\pm }/L^{3}$ are the ion densities. Typical simulations equilibrate for 4000τ, and data is accumulated over a 10,000 τ interval, where $\tau =\sigma {\left(m/\varepsilon \right)}^{1/2}$ is the MD time unit and the time step δt=0.005τ. Typical screenshots for different types of solvent affinities are presented in Figure 1. For comparison, we also consider an implicit solvent model at the some volume and temperature as our explicit solvent model, except that there is no solvent and all LJ interactions are described by Weeks–Chandler–Andersen potential.

## 4. Conclusions

In summary, we have investigated the influence of the solvent affinity for the ionic species on the conformational properties of flexible polyelectrolytes. Specifically, we focus on differences between the interactions between the solvent particles and the counter-ions, and the interactions between the solvent particles and the polyelectrolyte segments. We find that an enhancement of the solvent for either these two ionic species results in *qualitatively* similar trends in the impact of the polyelectrolyte chain size, and the radius of gyration of the polymer remains correlated with the size of the counter-ion cloud that surrounds the polymer chain. A *quantitative* comparison, however, indicates significant deviations. This effect was clearly revealed by considering the Pearson correlation coefficient between the radius of gyration of the polyelectrolyte chain and the net ionic charge at a given distance from the polyelectrolyte segments. In particular, we find that the Pearson correlation coefficient is positive at a short distance, but at larger distances becomes negative. Between the two types of solvent affinity, the Pearson correlation changes significantly, suggesting that an explicit description of the solvent in the theoretical treatment of polyelectrolyte solvents and gels is necessary. We conclude that the solution of ions and polymer chains significantly affects the resulting distributions of ionic species and polymer conformational properties in the solution, implying that a realistic modeling of polyelectrolytes must include an explicit solvent.
